# Supplementary material for: Superstability of micrometre jets surrounded by a polymeric shell
Source: J Appl Crystallogr. 2025 Jul 16;58(Pt 4):1261–8. doi: 10.1107/S1600576725004790 (PMC12321019; doi:10.1107/S1600576725004790)
Supplement: Supplementary file 1 [file j-58-01261-sup1.pdf]

JOURNAL OF
APPLIED
CRYSTALLOGRAPHY

Volume 58 (2025)

Supporting information for article:

Superstability of micrometre jets surrounded by a polymeric shell

A. Rubio, J. M. Montanero, M. Vakili, F. H. M. Koua, S. Bajt, H. N. Chapman and A. M. Gañán-Calvo

The videos `yr5155sup2.gif` and `yr5155sup3.gif` show images of a water jet and a protein microcrystal/aqueous buffer jet, each surrounded by the viscoelastic shell in a 0.6 mbar environment. The images (pixel size is 0.97 μm) were obtained with N9 for Q_i = 5 μl/min, Q_o = 12 μl/min and $m'_\mathrm{g} = 16$ mg/min. The crystal concentration was adjusted to be $c_\mathrm{crys} \simeq 15 - 20\%$ (*v*/*v*) after settlement.
