# Supplementary figures and images for: Superstability of micrometre jets surrounded by a polymeric shell
Source: J Appl Crystallogr. 2025 Jul 16;58(Pt 4):1261–8. doi: 10.1107/S1600576725004790 (PMC12321019; doi:10.1107/S1600576725004790)

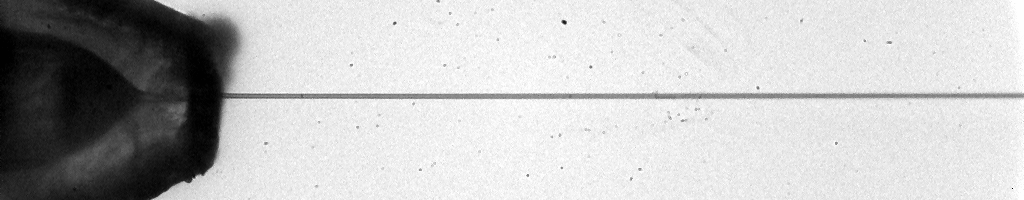

Supplement: Supplementary file 2 [file j-58-01261-sup2.gif]

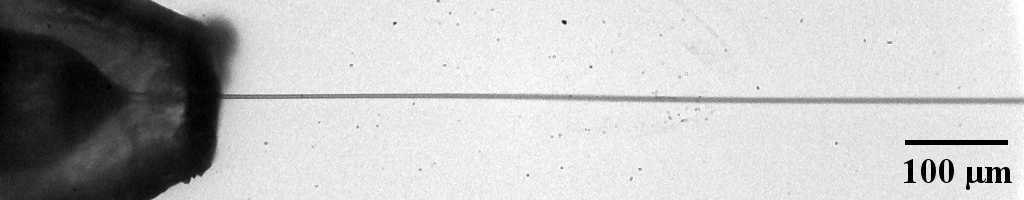

Supplement: Supplementary file 3 [file j-58-01261-sup3.gif]
